# Targeting Neural Hyperactivity as a Treatment to Stem Progression of Late-Onset Alzheimer’s Disease

**DOI:** 10.1007/s13311-017-0541-z

**Published:** 2017-05-30

**Authors:** Rebecca P. Haberman, Audrey Branch, Michela Gallagher

**Affiliations:** Department of Psychological and Brain Sciences, The Johns Hopkins University, 3400 North Charles Street, 116 Dunning Hall, Baltimore, MD 21218 USA

**Keywords:** Cognitive aging, Levetiracetam (Keppra), Late-onset Alzheimer’s disease, Hippocampal CA3, Amnestic mild cognitive impairment (aMCI)

## Abstract

Sporadic late-onset Alzheimer’s disease (LOAD), the most common form of dementia in the elderly, causes progressive and severe loss of cognitive abilities. With greater numbers of people living to advanced ages, LOAD will increasingly burden both the healthcare system and society. There are currently no available disease-modifying therapies, and the failure of several recent pathology-based strategies has highlighted the urgent need for effective therapeutic targets. With aging as the greatest risk factor for LOAD, targeting mechanisms by which aging contributes to disease could prove an effective strategy to delay progression to clinical dementia by intervention in elderly individuals in an early prodromal stage of disease. Excess neural activity in the hippocampus, a recently described phenomenon associated with age-dependent memory loss, was first identified in animal models of aging and subsequently translated to clinical conditions of aging and early-stage LOAD. Critically, elevated activity was similarly localized to specific circuits within the hippocampal formation in aged animals and humans. Here we review evidence for hippocampal hyperactivity as a significant contributor to age-dependent cognitive decline and the progressive accumulation of pathology in LOAD. We also describe studies demonstrating the efficacy of reducing hyperactivity with an initial test therapy, levetiracetam (Keppra), an atypical antiepileptic. By targeting excess neural activity, levetiracetam may improve cognition and attenuate the accumulation of pathology contributing to progression to the dementia phase of LOAD.

**Electronic supplementary material:**

The online version of this article (doi:10.1007/s13311-017-0541-z) contains supplementary material, which is available to authorized users.

## Introduction

Sporadic late-onset Alzheimer’s disease (LOAD), the most common form of dementia, is heralded by an insidious and progressive erosion of episodic memory in the later decades of life. Longitudinal observational studies have confirmed the existence of a symptomatic prodromal phase of LOAD, referred to as amnestic mild cognitive impairment (aMCI), in which memory deficits are greater than would be expected for a person’s age but do not significantly impair activities of daily life. This condition substantially increases risk for progression to the protracted phase of Alzheimer’s disease (AD) dementia during which deterioration continues until all cognitive domains are severely compromised [1, 2].

Investigations using disease biomarkers have now identified a prolonged asymptomatic stage of LOAD in which the accumulation of extracellular amyloid plaques can be detected by positron emission tomography (PET) neuroimaging in elderly individuals who are clinically normal [3]. Thus, the hallmark accumulation of pathological plaques containing aggregated forms of amyloid β (Aβ) peptide occurs prior to the advancing spread of neurofibrillary tangles composed of hyperphosphorylated tau and widespread neurodegeneration. Preclinical amyloid deposition, alongside the discovery of genetic links between Aβ mutations and risk for early-onset familial AD (FAD) [4, 5], has focused biomedical research and therapeutic strategies on reducing or reversing Aβ deposition. However, several high-profile failures of LOAD clinical trials targeting Aβ in patients with early- to mid-stage dementia have repositioned the timing of Aβ intervention to earlier disease stages and broadened the scope of therapeutic strategies to include alternative stage-specific targets [6–8]. Despite the fact that the clinical diagnosis of LOAD typically peaks in the seventh and eighth decades of life, it is often overlooked in therapeutic development programs that *aging itself* constitutes the single greatest risk factor for LOAD [9]. The aging context in which the long preclinical phase of pathophysiological development is situated has promoted greater attention to mechanisms by which brain aging may confer risk for progression of underlying pathology and symptomatic disease.

An emerging view of LOAD is that age-dependent alterations in neuronal activity act to promote the progression of memory loss and the accumulation of AD pathology. While neuron loss and hypoactivity are key features of later disease stages [10, 11], human neuroimaging studies, including those in elderly patients with aMCI, have identified a paradoxical hyperactivity signature in the hippocampus [12–16]. Initially thought to be a compensatory mechanism to support memory function, studies in elderly individuals and patients with aMCI have demonstrated that hippocampal hyperactivity is actually correlated with reduced cognitive performance within those populations [14, 17–20]. Significantly, pharmacological reduction of this overactivity in aMCI has demonstrated that it contributes to memory impairment rather than serving a beneficial role [20, 21]. Animal models of normal aging have also indicated that hippocampal hyperactivity is associated with memory impairment and that its natural absence in aged cohorts coincides with preserved cognition and pharmacological reversal in aged animals with cognitive impairment restores memory function [22–26].

Transgenic models designed to recapitulate the hallmark pathological features of AD have increasingly implicated excess neural activity as a causal and/or permissive factor in initiation and progression of Aβ and tau pathology. Young mice overexpressing human amyloid based on the genetics of early-onset FAD have shown that neuronal circuits become hyperactive in early stages of amyloid accumulation, contributing to neuronal injury [27–29]. Reduction of neural activity in such models produces beneficial effects on synaptic dysfunction and reduces amyloid deposition in some AD models [30]. Moreover, recent evidence from AD models of human tau mutations has demonstrated that chronically increased neural activity stimulates the release of tau and enhances the spread of tau pathology in the hippocampus and associated circuits [31]. Together with the emergence of circuit-specific hyperactivity in aging, these studies suggest that alterations in neural excitability may constitute an underlying basis that contributes to the risk of aging in late-onset AD progression.

In this review, we present evidence from both human clinical studies and animal models linking age-related hyperactivity with the worsening cognitive impairment found in early disease stages, as well as the accumulation of AD neuropathology. Further, we discuss therapeutic strategies for targeting age-related hyperactivity in early AD, with a focus on low-dose levetiracetam (LEV), an atypical antiepileptic that has shown efficacy in selectively reducing aberrant, but not basal, neural activity, resulting in improved cognitive outcomes in preclinical and clinical studies. Together these findings provide support for the hypothesis that aberrant neural activity in the aging brain may represent an underlying basis of risk for late-onset sporadic AD, and is a potential therapeutic target for delaying or preventing disease progression in the earliest stages of LOAD prior to clinical dementia.

## Hyperactivity in the Hippocampal Memory System is Localized to Key Computational Circuits in Normal Aging

The aging-specific, biological phenotype that confers risk for LOAD is difficult to separate from pathological processes in elderly humans owing to the lengthy preclinical phase of the disease superimposed upon an aging brain [3] and substantial variability in cognitive trajectories/outcomes across the aging human population. Studies of primates and rodents that do not evince overt AD pathology are informative for isolating changes due to brain aging apart from the pathological processes in AD. Consistent with individual differences evident in the normal aged human population, memory assessment also reveals individual differences in aged animal populations [32–34]. In aged rodents and monkeys with memory impairment, hyperactive neurons with elevated firing rates have been specifically localized to the CA3 subregion of the hippocampus in a manner consistent with the degree of memory impairment [23, 35]. In that context, studies using functional magnetic resonance imaging (fMRI) with high neuroanatomical resolution within the medial temporal lobe (MTL) have similarly localized excess age-related activation during memory performance to the dentate gyrus (DG) and CA3 (DG/CA3) subregions of the hippocampal formation in humans [18]. That excess activation detected by fMRI, as discussed further below, is also correlated with the degree of memory impairment in the elderly.

**Fig. 1:**
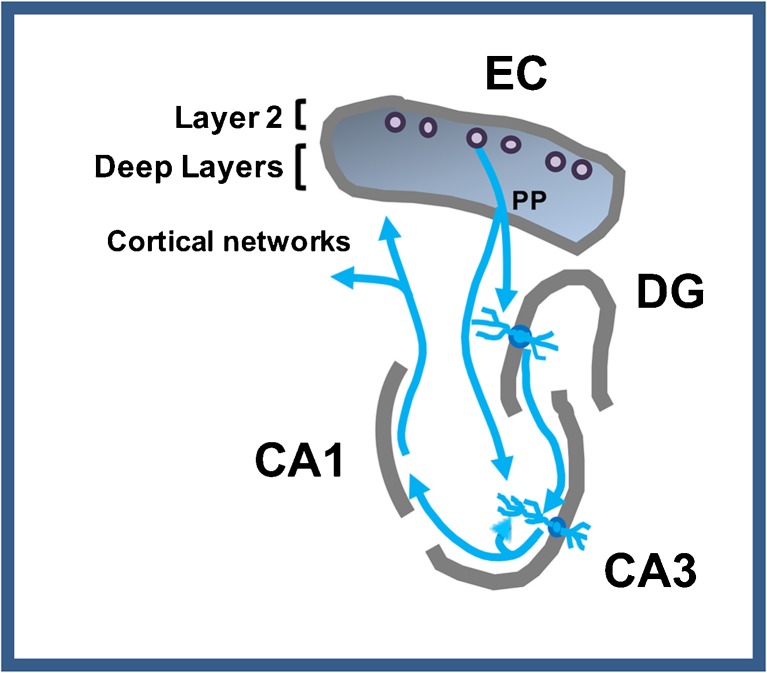
Schematic of connectional pathways. The perforant path (PP) from entorhinal cortex (EC) layer 2 cells provides innervation of the dentate gyrus (DG) and CA3. CA3 pyramidal neurons also receive synaptic input from DG mossy fibers and CA3 recurrent collaterals. The output of the DG/CA3 computational processing occurs through the CA1 subfield and the subiculum (not shown) projecting to deep layers of the EC and additional cortical structures

The contribution of the MTL system to episodic memory critically depends on the computational properties of the DG and CA3 subregions of the hippocampal formation of the MTL (Fig. 1). Layer 2 entorhinal cortex (EC) projections provide the primary input to the hippocampal network to encode the content of current experiences [36]. The normal function of the DG and CA3 subregions establishes distinctive representations in memory that minimize interference from similar experiences in the past. This ability to encode and retrieve information tied to specific events and experiences is critical for proficient episodic memory. The minimization of interference is normally implemented in the DG by “pattern separation”, referring to the highly distinctive encoding of input in a sparse network of granule cells, even when the input pattern has overlapping elements with prior information [37]. Encoding in the CA3 region, which receives its input from the DG, is also influenced by its strong autoassociative network, which retrieves similar representations from prior encoding by a process referred to as “pattern completion” [38]. These competing yet complementary processes are thought to minimize interference while increasing storage capacity for episodic memories [39, 40]. Computational models and empirical studies on the normal function of these circuits in laboratory animals have translated to normal young adults using neuroimaging methods [41, 42]. As reviewed in further detail elsewhere [43, 44], evidence for an effect of aging on the functional properties within this network occurs in age-related memory impairment in nonhuman species, as well as humans.

In aged rodents with memory impairment the balance of pattern separation and pattern completion within this network is shifted to diminish pattern separation in favor of pattern completion. Specifically, the encoding properties of neurons in this network of memory-impaired aged rats fail to rapidly develop distinctive representations that reflect pattern separation but instead exhibit interference in the retrieval of prior representations [26, 35]. Elevated neural activity directly recorded from CA3 neurons in aged memory-impaired animals in these studies likely contributes to this dysfunction in the balance of computational properties of the network [43]. Similarly, localization of excess fMRI activation in DG/CA3 region is observed in elderly humans, and likely also contributes to heightened interference in episodic memory [42, 44].

In tasks specifically designed to capture the key computational functions just described in humans, aged individuals are prone to mnemonic interference, exhibiting a shift in memory performance attributable to DG/CA3 dysfunction similar to that described in aged memory-impaired animals. To assess the capacity for pattern separation in humans, a recognition memory task using 3 judgments has now become widely used in neuropsychological research on aging and prodromal AD [45, 46]. In one common version of this task subjects are asked to judge whether pictures of everyday objects presented in a series are “new” (viewed for the first time), “old” (a repeat of a previous item in the series), or “similar” (resembling but not identical to a previous item in the series). A correct response of “similar” requires pattern separation to reduce mnemonic interference. Compared with young adults, older adults have less proficiency in the correct identification of items that are “similar” to a previously viewed item, instead calling such items “old”, an error indicative of a shift towards pattern completion [46–49]. High-resolution fMRI conducted during performance of the 3-judgment task showed greater hippocampal activation in the DG/CA3 region in elderly subjects compared with young adults alongside significantly worse performance, with fewer correct responses of “similar” and greater incorrect responses of “old” on those items [18]. Moreover, worse discrimination performance was significantly correlated with relatively greater DG/CA3 hippocampal activation.

**Fig. 2:**
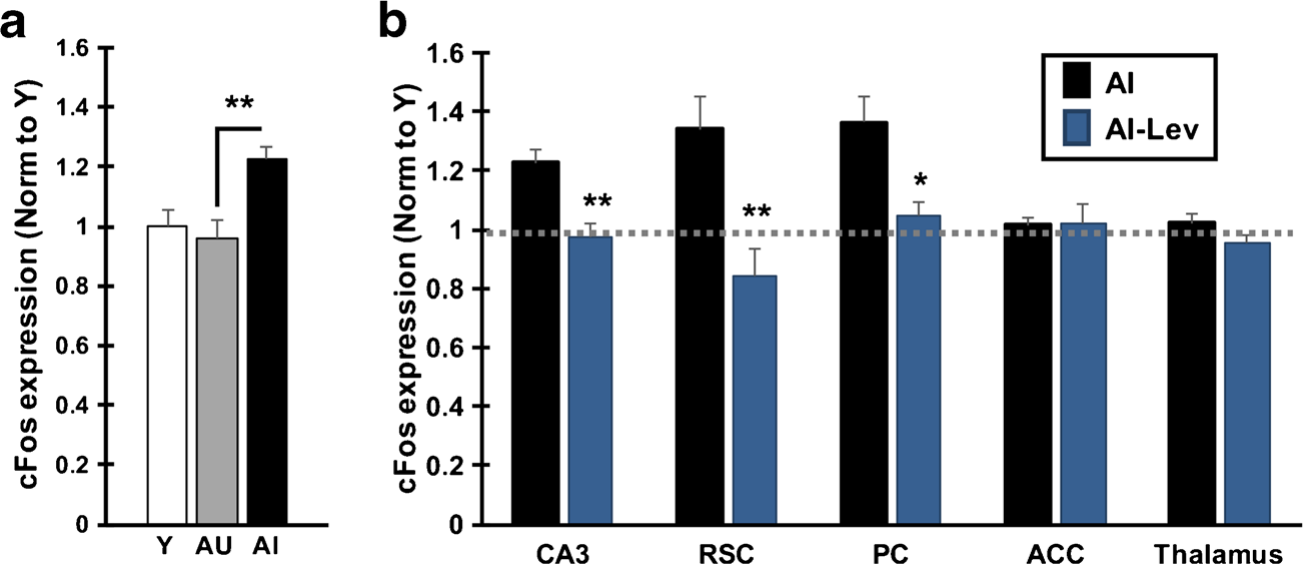
Pharmacologically induced neural activity is elevated in aged rats with memory impairment and reduced by levetiracetam (LEV) treatment. Neural activity was pharmacologically induced (pilocarpine, 25 mg/kg i.p.) in young (Y), aged rats with intact memory (aged unimpaired, AU) and aged impaired rats (AI). Induced neural activity was detected by quantification of cFos mRNA by *in situ* hybridization of brain sections collected 1 h after induction. (A) CA3 subfield of the hippocampus shows higher expression of cFos in AI rats compared with Y and AU rats. cFos expression also correlates with a measure of memory impairment among all aged rats (Pearson *r* = 0.832). AI rats also showed increased cFos in retrosplenial (RSC) and parietal cortex (PC) relative to both AU and Y rats (data not shown). (B) AI rats in the LEV condition (AI-LEV) were treated for 1 month prior to pilocarpine administration with LEV via osmotic pumps (10 mg/kg/day). Treatment with LEV reduced cFos expression in AI rats in the CA3 region of the hippocampus and select interconnected regions, including RSC and PC. ACC and thalamus show no impairment-dependent elevation of cFos and no reduction with LEV treatment. Significant difference across groups was determined by 1-way analysis of variance. Post-hoc significance was determined by *t* test as indicted on the graphs: *p < 0.05; **p < 0.01. Values represent group means ± SEM. Gray dotted line represents cFos expression in young rats. Figure adapted from *Neurobiology of Aging*, Haberman, RP, Koh, MT, and Gallagher, M, Heightened Cortical Excitability in Aged Rodents with Memory Impairment, in press**,** Copyright 2017) and reprinted with permission from Elsevier [22]. ACC = anterior cingulate cortex

A number of the studies assessing mnemonic interference in cognitively normal young and aged adults have highlighted individual differences in the elderly population. Notably, the subgroups of aged individuals with less proficient performance in 3-judgment recognition have corresponding differences in performance on standardized memory testing for delayed recall [46]. Individual differences are also common in aging across species. As a model of memory loss in normal aging, outbred rats show variability in cognitive outcomes such that a subgroup shows memory decline relative to young performance, while other aged cohorts exhibit preserved performance on par with young [33, 50]. These subgroups of “impaired” and “unimpaired” aged rats also differ in the encoding characteristics of neurons in the DG/CA3 network as already described. In recordings from ensembles of hippocampal neurons, similar to young adult rats, aged rats that are behaviorally characterized as unimpaired in memory performance exhibit rapid encoding of distinctive representations. In contrast memory-impaired aged rats, unlike young adult and unimpaired aged rats, fail to rapidly encode distinctive representations for new experiences, instead activating prior representations [26]. Individual differences in the heightened CA3 excitability associated with memory impairment in this model is evident by direct neural recordings [35]. This finding was replicated in a recent study using a pharmacological stimulus to induce neuronal activation and detection of activity by expression of the immediate early gene, cFos (Fig. 2A) [22]. cFos was increased in aged memory-impaired rats relative to both young and aged unimpaired rats, exhibiting a tight correlation with memory status such that greater cFos expression in CA3 coincided with poorer memory among the animals in the aged cohort.

In summary, memory impairment in both aged rats and aged humans is associated with neuronal hyperactivity similarly localized to the DG/CA3 subfields, with evidence from cognitive assessment indicating corresponding shifts in the balance of computational functions in hippocampal processing.

## An Augmented Detrimental Effect on the Computational Functions of the Hippocampal System in aMCI

Numerous studies have now demonstrated that hippocampal hyperactivity is augmented in patients with aMCI when compared with age-matched control subjects [10, 15, 20, 21, 51–55], and is a highly consistent and characteristic signature in aMCI. This hyperactivity is also a measure that predicts subsequent cognitive decline/conversion to a dementia diagnosis [12–14] and is significantly correlated with the extent of neuronal injury affecting AD-specific regions of the aMCI brain [17]. Moreover, hippocampal hyperactivity is most pronounced in MCI associated with AD pathology, as determined by PET amyloid imaging [56]. Furthermore, that hyperactivity persists in the MCI phase of the disease over a 3-year follow-up, during which time greater clinical/cognitive worsening is evident in amyloid positive patients with MCI, relative to patients with amyloid negative PET scans.

**Fig. 3:**
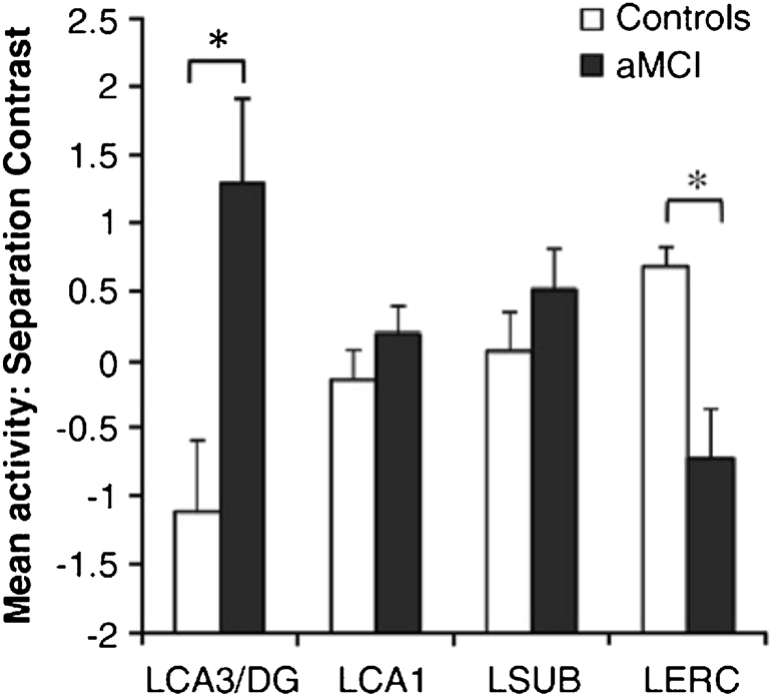
High-resolution functional magnetic resonance imaging signals in amnestic mild cognitive impairment (aMCI) *vs* age-matched controls. Patients with aMCI had significantly higher task-related activation in the left CA3 (LCA3)/dentage gyrus (DG) together with lower activity in the left entorhinal cortex (LERC) during encoding for similar items on 3-judgement task. * aMCI significantly different from controls. Figure reprinted from *Neuroimage*, 51, Yassa, MA, Stark, SM, Bakker, A, Albert, MS, Gallagher, M and Stark, CEL, High-resolution structural and functional MRI of hippocampal CA3 and dentate gyrus in patients with amnestic Mild Cognitive Impairment, 1242-1252, Copyright (2010) with permission from Elsevier [15]. LCA1 = left CA1; LSUB = left subiculum

Using high-resolution fMRI to localize differences in activation within the MTL in prodromal AD, studies have consistently reported elevated activation in the DG/CA3 subregions in aMCI relative to age-matched cognitively normal controls (Fig. 3) [15, 20, 21, 54]. Together with that regionally defined augmentation of fMRI activation, the profile in age-related memory impairment in elderly humans is also significantly magnified in patients with aMCI. Relative to age-matched controls, the performance of patients with aMCI on 3-judgment tasks shows a further worsening in the ability to make correct responses to “similar” items while committing more errors in identifying such items as “old” [20, 21, 46]. The further heightening of fMRI activation in the DG/CA3 subregions of the hippocampus is tightly correlated with this worsening performance [15, 20].

The clinical condition of aMCI is characterized by a symptomatic worsening of memory performance beyond that considered normal in the aging population. The studies just described in aMCI tie this condition to alterations in the specific circuits and cognitive processes that are also vulnerable in aging. These commonalities may indicate that age-related memory impairment and the transition to the early symptomatic phase of AD exist on a continuum. In that context, a recent report directly links the existence of hyperactivity to the accumulation of AD pathology in humans. In the study by Leal et al. [57] cognitively normal elderly subjects received neuroimaging with fMRI at baseline and were followed for 3 years with longitudinal PET imaging for amyloid. Hippocampal hyperactivity in the study group at baseline predicted the accumulation of AD pathology.

## Reduction of Hippocampal Hyperactivity with LEV has Therapeutic Efficacy in Aging and aMCI

The functional significance of the association between hippocampal hyperactivity and MTL-dependent memory deficits in aging and aMCI was initially subject to differing interpretations. According to one view, elevated hippocampal activity detected by fMRI during mnemonic tasks was interpreted as a compensatory mechanism to support memory function by recruiting a greater response in a failing network [10]. Alternatively, increased activation might reflect a dysfunctional condition contributing to memory impairment by shifting hippocampal computational processing as described above. Experimental evidence subsequently obtained by targeting hippocampal hyperactivity with treatments that reduce activity demonstrated support for the latter view that overactivity, detected by fMRI in humans and multiple measures of elevated neural activity in animals, contributes to memory impairment.

### Targeting Hyperactivity Improves Memory Performance in Aged Rats

Research in aged cognitively-impaired rats first tested the hypothesis that CA3 hyperactivity served to impair cognition. Koh et al. [24] targeted CA3 hyperactivity both locally, using virally transduced inhibitory neuropeptides, and systemically, with peripheral administration of low-dose atypical antiepileptics, valproate, and LEV. Both local and systemic treatments resulted in cognitive improvement in aged impaired rats on 2 independent spatial memory paradigms that critically depend on intact MTL function. A LEV dose–response curve demonstrated efficacy in a low-dose range (5 and 10 mg/kg), significantly lower doses than required for antiepileptic efficacy in seizure models.

The cognitively effective dose of LEV that restored memory in aged impaired rats has been used in other protocols that demonstrate heightened excitability of hippocampal neurons. Pharmacological induction of neural activity detected by elevated cFos expression in the hippocampal CA3 subfield in aged rats with memory impairment is reduced by LEV treatment (Fig. 2B). With LEV treatment, cFos expression in the aged impaired rats remained similar to levels expressed in young rats and aged rats with preserved cognition [22]. In addition, multiunit recording of hippocampal CA3 neurons also showed reduction of neuronal firing rates in response to targeting overactivity with systemic drug treatment [58]. These investigations demonstrate that targeting aberrant excess hippocampal activity is an effective strategy for improving memory in aged rats with impairment.

A second effective preclinical approach to targeting overactivity in aged impaired rats has focused on the use of positive allosteric modulators of γ-aminobutyric acid (GABA)-Aα5 receptors. These receptors normally mediate tonic inhibition and have exceptionally high expression localized to the hippocampus relative to other brain regions [59]. Negative allosteric modulators of GABA-Aα5, which increase hippocampal excitability, were previously reported to modestly increase performance of young rats in spatial memory paradigms [60–63]. In contrast, the use of a negative allosteric modulator showed no such beneficial effect in memory-impaired rats with a condition of hippocampal overactivity. Instead, positive allosteric modulators of GABA-Aα5 receptors, which reduce neural excitability, have been shown to improve memory performance in aged memory-impaired rats [25]. The high expression of GABA-Aα5 receptors on CA3 pyramidal neurons makes them ideally positioned to control the overactivity of those neurons. In addition, recent experimental evidence has demonstrated a contribution of GABA-Aα5 receptors in the DG to pattern separation [64]. Removal of DG GABA-Aα5 receptors reduced tonic, but not phasic, inhibition and elevated the response in DG to a stimulating input. Elevated DG activity was coupled to impaired performance on behavioral tests of pattern separation, consistent with a contribution of hyperactivity to impaired DG/CA3 computational processing.

Together, these findings support the conclusion that elevated hippocampal activity serves to impair hippocampal function in aged rats. While the use of selective GABA-Aα5 positive allosteric modulators are a promising therapeutic approach currently in preclinical development, given the long history of LEV safety at much higher doses to treat patients with epilepsy [65–67], this treatment was used in clinical studies of patients with aMCI to determine whether targeting hippocampal hyperactivity would show translational evidence for benefit.

### LEV Reduction of Hippocampal Hyperactivity in aMCI Improves Memory Performance

Successful treatment in aged impaired rats led to clinical testing of LEV in elderly patients with aMCI [20]. Mechanistic studies of LEV action suggest it is a promising proof-of-concept drug to examine the clinical outcomes of reducing excess activity. By binding to the synaptic vesicle protein, SV2a, LEV selectively dampens neurotransmitter release under conditions of elevated firing, but not baseline neurotransmission [68–70].

To assess the effect of LEV on elevated activity and behavior in patients with aMCI, a low dose (125 mg q12h), equivalent to an efficacious dose in rodents, was initially tested. A double blind, within-subject crossover design allowed the comparison of an individual’s memory performance with and without LEV treatment [20]. Memory was tested in the 3-judgement memory task as previously described, and concurrent fMRI scans confirmed task-specific excess activation of DG/CA3 subdivision of the hippocampus. LEV treatment significantly reduced DG/CA3 activation in patients with aMCI and significantly improved their performance on the 3-judgment memory task, increasing correct responses of similar (indicative of improved pattern separation) while committing fewer errors of “old” responses to those items. The improvement of patient performance under treatment that reduced hippocampal hyperactivity provided the first evidence that fMRI overactivity in the hippocampus is not a beneficial signature for compensatory function but rather represents a condition contributing to impairment.

Using a range of dosing regimens in independent cohorts of patients with aMCI, LEV’s ability to reduce hyperactivity in the hippocampus with improvement in memory task performance was confirmed [21]. Notably, LEV only improved task performance at doses that reduced the elevated fMRI signal in DG/CA3 [21]. The three cohorts of patients were treated with different doses of LEV, which were selected to achieve brain exposures similar to those shown to have efficacy (low doses) or lack of efficacy (high dose, 250 mg q12h) in preclinical models. In the within-subject crossover design, each aMCI cohort on placebo exhibited significantly increased DG/CA3 hippocampal activation when compared with healthy age-matched controls and was similarly impaired in task performance relative to normal aging. Low doses in the range of efficacy observed in the preclinical research significantly improved memory task performance and normalized fMRI signals so that no difference remained in comparison with age-matched controls. In addition, similar to the results observed in memory-impaired aged rats, therapeutic efficacy was lost at a higher drug exposure that remains below the range used therapeutically in epilepsy. These data provide strong evidence that LEV reduction of excess neural activity in the hippocampus provides therapeutic benefit and demonstrates that hippocampal hyperactivity contributes to impaired memory rather than performing a compensatory role.

It has long been recognized that the MTL system is vulnerable both in aging when memory complaints become quite common and also in the early pathological progression of AD. The fact that both aging and AD involve common specific circuits within this system has become better defined in relatively recent research. It is not yet clear, however, whether these parallels reflect an underlying high vulnerability of specific circuits to a variety of conditions acting independently, aging on the one hand and the pathophysiological insults of AD on the other, or whether the condition of aging itself contributes to the vulnerability and progression of disease [71].

## Neural Hyperactivity and AD Pathophysiology

As discussed in previous sections, heightened neural activity is found in both normal aging and in the preclinical and aMCI phases of AD. Transgenic rodent models of AD have been created and studied by overexpressing rare human mutations in the amyloid precursor protein (APP) and associated secretase components (Psen1 and Psen2) identified in early-onset FAD and tau mutations causal for frontotemporal dementia [72, 73]. While rodents do not naturally develop AD pathology, the exposure of the murine brain to high levels of Aβ results in AD-like changes, including amyloid plaques, neuritic dystrophy, gliosis, synaptic deficits, and a range of cognitive and noncognitive behavioral alterations [74–80]. Similarly, mouse lines carrying frontotemporal dementia tau mutations display neuronal and synaptic dysfunction, inflammatory responses, and axonal degeneration [81–85]. While no AD model perfectly recapitulates the spectrum of human AD progression, these models provide an opportunity to identify the contribution of individual pathological disease factors *in vivo*, as well as settings for proof-of-concept tests of therapeutic strategies. Increasing evidence from such models indicates an interrelationship between AD pathology and neural activity, strongly supporting hyperactivity as central to the pathological mechanisms of Aβ and tau.

Most mouse models for AD are based on the amyloid hypothesis of AD progression (reviewed in [86]). The major protein component of amyloid plaques found in AD is a small 39 to 42-amino acid polypeptide called Aβ, which is derived from the proteolytic cleavage of the transmembrane localized APP [5]. Aβ cleavage results in its release into the extracellular space where it interacts with a variety of receptors, and when present at high levels, aggregates into toxic amyloid plaques. Numerous mutations have been identified in APP and enzymes which regulate its cleavage, the molecular details and effects of which have been extensively reviewed elsewhere [4, 5]. Pathogenic mutations associated with FAD cause either an increase in the total levels Aβ, or modify its cleavage such that it is biased towards its longer form Aβ_42_, which is more strongly associated with the formation of plaques than the shorter Aβ_40_ [4, 87, 88]. Transgenic mouse models based on these mutations have high levels of Aβ and develop amyloid plaque pathology similar to that seen in human AD, and display behavioral deficits in learning and memory tests [73, 89].

Evidence for hyperactivity associated with Aβ was first documented in transgenic mouse models of AD that overexpress human mutant APP via strong exogenous promoters. EEG recording in freely behaving young adult hAPP-J20 mice showed widespread elevated, but subconvulsive activity in the hippocampus and cortex, which was accompanied by impaired synaptic plasticity mechanisms and other evidence of neuronal injury [77, 90]. Epileptiform activity has since been observed in several transgenic models [91–93], as have spontaneous epileptic seizures [94, 95], which are attributable to increased network hyperexcitability [91, 96]. The relationship between this aberrant activity and Aβ began to be elucidated in separate studies of plaque-bearing double transgenic mice (APP23 × PS45) mice carrying APP and PSEN1 mutations where increased numbers of hyperactive neurons were identified in proximity to amyloid plaques across cortical networks including the frontal cortex [97], primary visual cortex [98], and the hippocampal CA1 subfield [27], suggesting that Aβ aggregates may induce higher neural activity. This phenomenon was further confirmed in APPswe/PS1d9 animals, carrying slightly different mutations, using activity dependent reporters [99] and whole-cell patch clamp recordings to demonstrate elevated neural activity alongside dendritic abnormalities [100]. Intriguingly, a subsequent study of APP23 × PS45 mice indicated that the number of hyperactive neurons present in the CA1 of the hippocampus was substantially elevated at an age prior to plaque deposition relative to controls [27]. This heightened activity was blocked in transgenic animals following reduction of soluble Aβ levels with a γ-secretase inhibitor, and could be induced in wild-type animals by local application of soluble Aβ oligomers, providing strong evidence for a role of soluble Aβ species as the primary toxic driving force for this hyperactivity, rather than already deposited plaques [29].

The hyperactivity signature associated with familial genetics in these animal models and early-onset FAD may differ in some respects from that observed in aging and prodromal LOAD. A condition of pathological neural hypersynchrony is associated with elevated network epileptiform activity in several of the FAD mouse models. Aberrant epileptiform activity, possibly driven by this hypersynchrony, also appears to be more common clinically in early-onset FAD [101, 102] than in LOAD [103]. A basis for heightened neural activity associated with aging that is nonepileptiform would be consistent with a lower risk for seizures in LOAD relative to early-onset FAD. Nonetheless, irrespective of such differences, evidence across the spectrum of AD suggests a bidirectional effect of Aβ and neural activity.

A recent study builds on the earlier findings that regulation of neural activity modulates Aβ production [29, 104]. Using viral-mediated expression of exogenous receptors (DREADDS) [105], Yuan et al. [30] introduced chronic intermittent increases or decreases in neural activity in APP-overexpressing mice. Strikingly, increases in Aβ levels and deposition were observed following elevation of neural activity, while activity reduction resulted in decreased Aβ accumulation, as well as reduced axonal dystrophy and synaptic loss in areas nearby amyloid plaques. While these studies do not exclude the possibility that other factors may contribute to the pathological effects of Aβ, they provide support for the existence of a feed-forward induction loop between Aβ and neural activity. This circular relationship suggests that either elevated neural activity or elevated Aβ may initiate a cascade towards increasing hyperactivity and peptide overproduction leading to amyloid deposition, and, importantly, that restoring balanced activity can ameliorate Aβ toxicity.

Similar to aged rats and patients with aMCI, therapeutic targeting of neural activity in AD rodent models has been demonstrated to improve cognition and signatures of neuronal injury. In the hAPP-J20 model, administration of LEV improved behavioral performance across several tasks [106], and reduced signatures of neuronal injury [90]. Additional behavioral and neurophysiological efficacy has been shown in other AD models in which amyloid is associated with neuronal hyperactivity [107–110]. Consistent with the LEV mechanisms of selectively targeting aberrant activity, the effects in mutant APP mice produced by LEV (e.g., brain markers and behavior) were not observed in control nontransgenic (nonamyloid) mice given the same drug treatment. Furthermore, a spectrum of other antiepileptic drugs, with mechanisms of action that differ from the atypical antiepileptic LEV, have been ineffective in APP models, including ethosuximide, gabapentin, phenytoin, pregabalin, and vigabatrin [106, 110]. In that context, it is also notable that doses of LEV that are effective *in vivo* in preclinical AD models have been consistently lower than those required for antiseizure efficacy in epilepsy models.

Many effects in mutant APP mice are prevented by removal of endogenous tau [86, 111]. The regulation of excitability is a leading explanation for the neuroprotection conferred by tau reduction, including a role for endogenous tau in early synaptic pathology and in the toxicity observed in mouse models based on the P301Ltau human tau mutation [112, 113]. In addition to limiting hyperactivity in mutant APP mice, the reduction of endogenous tau prevented cognitive impairment, synaptic/molecular dysfunction, and neuronal injury in a manner similar to low-dose LEV treatment [90, 93, 106, 111]. More recently, tau was shown to be required for an increase in hippocampal neuron dendritic excitability initiated by Aβ; here tau removal was also mimicked by LEV which prevented both potassium channel depletion and dendritic hyperexcitability [107]. Because a loss of synaptic integrity includes dendritic structural degeneration caused by hyperexcitability [100], such early neurodegeneration could be limited by LEV.

The biological evidence from AD mouse models supports a relationship between neural activity and AD pathology that is bidirectional. In that context, the hippocampal overactivity that develops with aging is a possible initiator and/or potentiator of pathological processes in LOAD, as supported by recent evidence in humans [57]. The circular relationship between activity and amyloid, in particular, may initiate a vicious cycle of increasing hyperactivity driving greater pathology, supporting the perspective that aging itself plays a role in contributing to vulnerability for disease.

## MTL System and Cortical Networks in Aging, AD Models, and aMCI

Biomarkers for preclinical AD have been detected in brain regions outside of the hippocampus proper, specifically in cortical regions of the MTL and circuits in the default mode network (DMN) that are strongly connected with the hippocampus. Beneficial effects of LEV have been reported not only on the targeted hyperactivity in the hippocampus, but extend to the broader MTL–cortical network in age-related impairment, as well as in mouse models of AD. This section highlights data that indicate certain parallels in the impairment-related alterations of the aging brain and vulnerability of specific sites to AD pathology within these extended MTL–cortical circuits, including beneficial effects of LEV treatment across a broad network.

### EC and MTL Circuitry

**Fig. 4:**
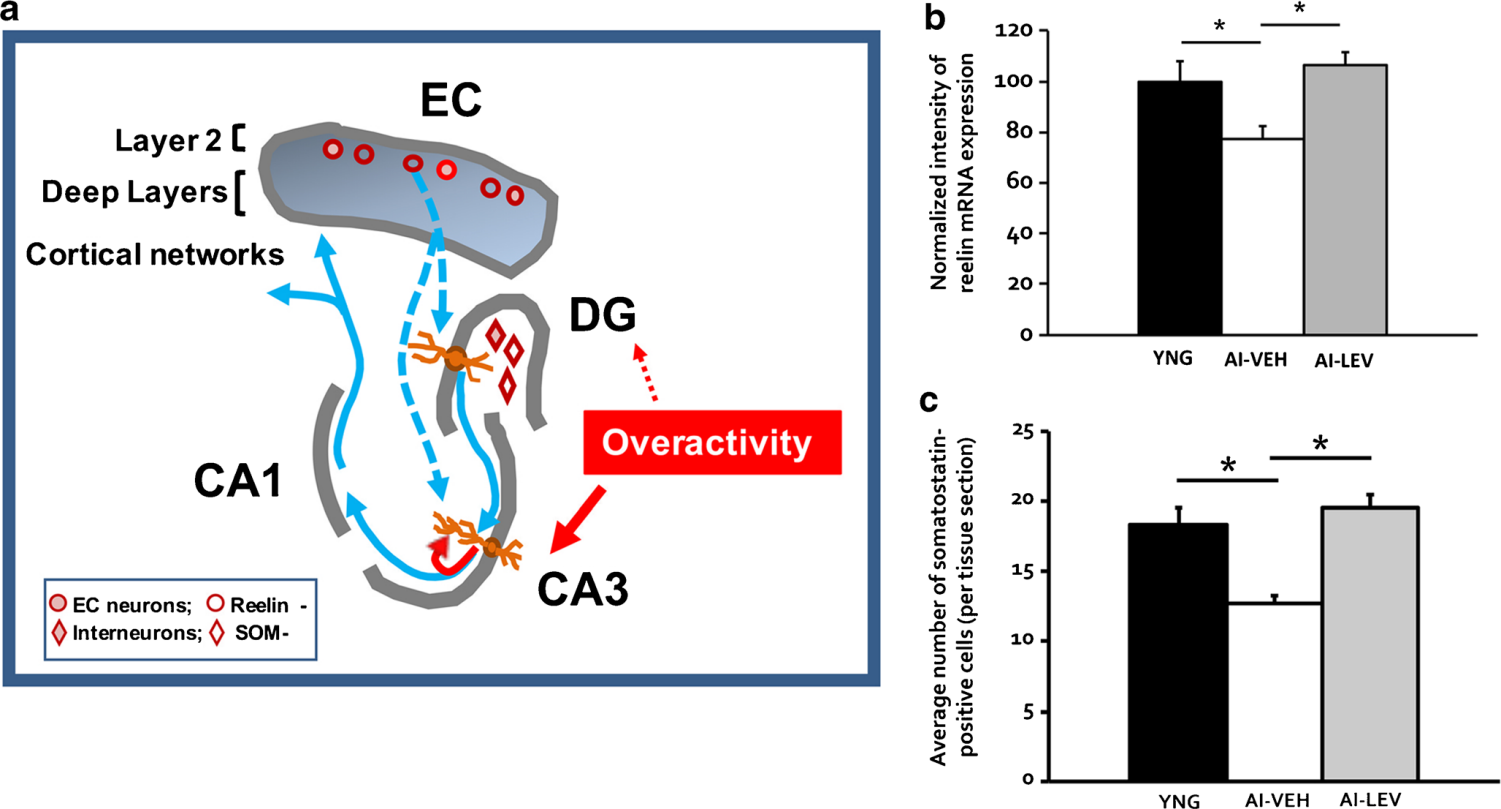
Alterations coinciding with hippocampal hyperactivity and memory impairment. (A) Schematic illustrating the reduction of Reelin expression in entorhinal cortex (EC) layer 2 and somatostatin (SOM) expression in the dentate hilus interneurons in age-related memory impairment. (B) These data replicate the findings in Stranahan et al. [122] of reduced reelin mRNA in EC layer II neurons of aged impaired (AI) rats and its reversal in AI rats treated with levetiracetam (LEV) by osmotic minipump (10 mg/kg/day) for 28 days (figure panel provided by Koh, MT and Gallagher, M); *p < 0.05. (C) Somatostatin expression in dentate hilus interneurons is reduced in AI rats and rescued by the same osmotic minipump treatment with LEV; *p < 0.05. Part (C) adapted from Spiegel, A.M., Koh, M.T., Vogt, N.M., Rapp, P.R., & Gallagher, M. Journal of Comparative Neurology vol 521, p. 3508-3523. Copyright (2013) reprinted with permission from John Wiley and Sons [128]. DG = dentate gyrus; YNG = young; AI-VEH = AI rats treated with saline vehicle; AI-LEV = AI rats treated with LEV

The layer 2 neurons of the EC form the perforant path input to the DG/CA3 subfields of the hippocampal network, as schematically illustrated in Fig. 4(A). The layer 2 EC neurons are distinguished for their vulnerability in AD, representing the earliest lesion affected by frank neurodegeneration. In autopsied brains well-characterized for amyloid and tau pathology, loss in the number of layer 2 EC neurons can be detected in the prodromal AD phase of MCI (clinical dementia rating (CDR) 0.5 [1]), while no loss is detected in normal aging in the absence of AD pathology [114]. In other species, including rodents and nonhuman primates, numbers of neurons in the EC, and in layer 2 specifically, are preserved, even in aged animals with behaviorally assessed memory impairment [115–117]. However, a loss in the integrity of these neurons is evident across the spectrum of age-related memory impairment in laboratory animals and humans. An example of this altered condition involves the expression of reelin, which is a phenotypic marker of layer 2 EC neurons across species [118, 119]. In the adult brain, strong evidence demonstrates reelin contributes to normal synaptic function and plasticity [120, 121].

In aged rats with memory impairment both mRNA and protein expression of reelin in layer 2 EC neurons is reduced, a condition that is rescued by LEV treatment [122]; Fig. 4B). In the brains of aged rhesus monkeys characterized for memory performance, reelin reduction in layer 2 EC is similarly observed in memory-impaired subjects with expression in aged unimpaired monkeys remaining on a par with young adults (P.R. Rapp, personal communication). Loss of EC reelin is also seen in mouse models of FAD and in patients with AD, consistent with the well-known vulnerability of neurons in layer 2 of the EC [123, 124]. Given evidence for reelin in regulating excitability and synaptic plasticity, its loss in layer 2 EC may alter not only the normal function of this key input pathway, but may contribute to the pathology of Aβ and tau in AD. Indeed, in reelin heterozygous mutant mice crossed with familial AD transgenic mice, reelin reduction accelerates amyloid and tau pathology in hippocampal circuits [125] and has been co-localized with intracellular Aβ in EC neurons in an AD rat model, as well as in early AD brains [126]. Recovery of reelin expression with LEV in aged subjects may indicate that this early-stage loss of neuronal integrity in the EC is a reversible condition.

As described in an earlier section of this review, a critical computation function on the EC input in the DG is to distinctively encode input patterns in a small number of granule cells. Mechanisms for sparse encoding in the DG are under local inhibitory control (illustrated in Fig. 4A). Selective targeting of DG inhibition can disrupt this function leading to greater neuronal activation by an input pattern and behavioral impairment on tasks with high mnemonic interference [64, 127]. In aged rats with memory impairment, stereological assessment of interneuron numbers found a reduction in a subpopulation of DG hilar inhibitory neurons that co-express somatostatin (SOM) relative to young rats and aged rats with unimpaired memory [128], while total numbers of neurons in the hilus were unchanged. This “HIPP” subpopulation, with cell bodies located in the hilus, innervates the molecular layer at the perforant path input from EC layer 2. Additional evidence for the relevance of SOM neuron loss in aging and risk for AD comes from studies of the human ApoE4 allele, a known risk factor for LOAD. ApoE4-positive individuals show elevated hippocampal activity during a memory task, suggesting this allele modifies excitatory/inhibitory balance [129]. Expression of the ApoE4 allele in mice exacerbated the age-dependent loss of hilar SOM neurons relative to the risk-neutral E3 allele [130]. Furthermore, E4 allele expression in this study contributed to greater memory impairment in older mice. The ApoE4 effect on SOM neurons potentially links aging and augmented risk for AD in carriers of the E4 allele. Notably, as shown in Fig. 4(C), similar to reelin expression in EC, SOM expression in HIPP interneurons in the hilus/DG was rescued by LEV treatment in memory-impaired aged rats [128].

In addition to these examples, other network-wide benefits of LEV treatment have been described in APP models of amyloid overexpression. As reviewed elsewhere [78, 103], AD pathology causes complex derangements of neuronal activity affecting extensive interconnected networks. Alongside aberrant excitatory activity, compensatory responses can also become engaged in an adaptive reorganization of the network around overactivity. A relatively widespread normalization in such network function has been observed by targeting hyperactivity in AD mouse models. For example, in hAPP-J20 mice treated with LEV, beneficial effects on synaptic plasticity in the DG were observed alongside a normalization of throughput, for example electrophysiological recording of input/output functions, at CA3/CA1 synapses [106]. Such evidence for a network effect of LEV extends to patients with aMCI, who, alongside hippocampal overactivity, exhibit reduced fMRI activation in the EC (Fig. 3A) that is likewise reversed with LEV [20, 21]. These data support a network perspective on both the condition of cognitive impairment and remediation of cognitive decline.

### Disruption of Function in Regions of the DMN

Altered neural function associated with AD pathology and aging also occurs in cortical networks functionally interconnected with the MTL. In AD mouse models, Aβ associated hyperactivity is closely tied to corticocortical and corticohippocampal circuit disruptions that are suggested to impair memory function [77, 131]. Likewise, human imaging studies show altered functional connectivity across brain regions in elderly humans and those with aMCI [132–134], including early accumulation of high levels of amyloid pathology particularly evident in component structures of the DMN [132].

The DMN is a distributed cortical network defined by coordinated activity in a resting state that shows deactivation (reduced activity) in favor of task-specific network activation during cognitive engagement. Posterior components of DMN, including precuneus, posterior cingulate, retrosplenial cortex, and bilateral inferior parietal lobule, which exhibit strong functional connectivity with MTL, have been earlier described as a hippocampal–parietal memory network [135]. Altered functional connectivity across the DMN and reduced “deactivation” is evident with aging and cognitive impairment in clinical studies [136–138]. For example, a recent study of cognitively normal older individuals reported reduced DMN deactivation, localized to posterior cortical regions of the DMN, that occurred specifically in a subgroup of individuals that experienced cognitive decline based on their own longitudinal performance on neuropsychological assessments compared with individuals who had maintained cognitive abilities [138].

We recently demonstrated that alongside the CA3, evidence for heightened excitability is also present in posterior cortical regions interconnected with the hippocampus in aged memory-impaired rats. In response to pharmacological induction of neural activity, aged impaired rats exhibited elevated levels of cFos, relative to young and aged-unimpaired rats, in a subset of the structures that comprise the rat DMN, including posterior parietal cortex and retrosplenial cortices. In the impaired aged rats, elevated cFos was limited to specific cortical regions, with no excess elevation in the anterior cingulate cortex or thalamus (see Fig. 2B) [22]. Important in the current context, the elevated posterior cortical activity in aged impaired rats responded to a cognitively effective dose of LEV treatment, lowering induced cFos to the level of young rats. Notably, no effect of LEV in the aged impaired subjects was seen in any region that did not show initial heightened activation.

MTL deficits are recognized as contributing to the cognitive impairment observed in both aging and aMCI. Interest in the impact of AD on distributed neocortical networks has gained increasing attention, particularly since neuroimaging has shown deposition of cortical amyloid in preclinical AD. Early amyloid deposition is observed in DMN, including regions that are strongly interconnected with the hippocampus and MTL network [132, 139]. Accumulated pathology has been associated with altered activity in MTL cortical regions interacting with the hippocampus in some studies [140].

In the case of cortical circuits, similar to the MTL, it is difficult to separate effects of aging from pathological processes in elderly humans owing to the lengthy preclinical phase of the disease superimposed upon an aging brain. Here, again, the use of a rodent model for age-related impairment, devoid of the natural occurrence, or genetically introduced, pathophysiology of AD, has shown potentially important parallels between aging and AD pathology in a broad MTL/cortical network. Specifically, the intervention with LEV in the model of age-dependent memory impairment suggests that an altered condition may exist underlying the vulnerability of those circuits to early AD pathology. While the use of *in vivo* assessment has been integral to illuminating the affected circuitry in aging, further work is needed to address whether treatment effects are obtained by direct action of therapeutics on numerous distributed sites or whether widespread normalization of network function reflects both primary and secondary effects of targeting hyperactivity. In either case, a network perspective is needed to address the basis for impairment and understanding the effects of therapeutic intervention in aging that may extend to the progressive cognitive decline and accumulation of pathology in early LOAD.

## LEV Mechanism of Action Supports Successful Use as Therapy

The atypical antiepileptic, LEV, was approved in 1999 as a second-generation drug and is most often used as an add-on therapy in the treatment of epilepsy [141]. Even at much higher chronic dosing as an adjunctive treatment in epilepsy, LEV has an excellent safety profile with few side effects in elderly patients [66, 142].

Mechanistically, LEV is well positioned to affect neuronal function in a beneficial manner in the condition of overactivity in aging and prodromal AD where efficacy has been reported with low-dose administration. LEV belongs to a class of compounds with high affinity for the presynaptic membrane protein SV2a, which is widely expressed throughout the brain, including high levels of expression in the hippocampus [143]. While the role of SV2a in biological function is not completely understood, strong evidence demonstrates a role in modulating calcium-dependent neurotransmitter release via multiple mechanisms with a greater effect during high activation [69, 144–146]. SV2A influences neurotransmitter release via expression and trafficking of the calcium sensor synaptotagmin and likely binds directly to synaptotagmin [145, 147]. SV2a also contributes to the mobilization of synaptic vesicles for release, and SV2a deletion reduces vesicle release during trains of action potentials but does not measurably affect steady-state activity [144, 146]. LEV treatment has shown a beneficial effect in modulating neurotransmission in a number *in vitro* and *in vivo* models of elevated activity, and the binding of LEV to SV2a is likely a primary mechanism of action [24, 106, 148].

Other beneficial effects of LEV have also been identified that improve neuronal dysfunction induced by AD pathology. In addition to mechanisms for quieting overactive neurons by limiting transmitter release, LEV has been demonstrated to inhibit both ryanodine and IP3 receptor-activated calcium release in hippocampal neurons [149, 150], which would be neuroprotective in the context of impaired calcium homeostasis in AD [151, 152]. Recent evidence of further interest has also shown beneficial effects of LEV on mitochondria, in which amyloid-associated mitochondrial dysfunction of fission and fusion imbalance was corrected by LEV [153]. In total, LEV treatment appears to have many beneficial effects on brain deficits associated with impaired cognitive function, induced by aging or AD pathology, with reduction of elevated neural activity most central to its effects.

## Targeting Hippocampal Hyperactivity to Stem Disease Progression

With the number of Americans living with AD dementia expected to more than double by 2050, it is predicted that development of a therapy that delays onset or slows progression to clinical dementia by even 1 to 2 years could reduce AD prevalence by 10% to 20% [154, 155]. In this review, we have presented evidence for the efficacy of targeting hippocampal hyperactivity using LEV as an initial therapeutic intervention. As a condition present in the earliest stages of AD, reduction of hyperactivity has strong potential to affect the course of the disease and its rate of progression. While studies in animal models of aging and a phase II trial in aMCI support the case for targeting hyperactivity, clinical testing in a phase III trial is needed to ascertain the long-term effects of this treatment approach on progression. An appropriate phase of disease for such treatment could be MCI due to AD, in which hippocampal hyperactivity is most pronounced. In addition to targeting hyperactivity as a monotherapy, given evidence for hyperactivity driving AD pathology, this treatment may also be useful in combination with other therapeutic approaches, such as amyloid-lowering therapies or treatments targeting tau.

## Conclusions

Based on the evidence presented, we propose a model of LOAD whereby aging itself creates an environment in which abnormal, elevated neural activity promotes circuit dysfunction and contributes to the accumulation of pathology. In this view, the emergence of hyperactivity in the hippocampus and alterations in associated MTL circuits are key early events in progressive neural dysfunction. Indeed, recent investigations indicate that hippocampal hyperactivity may occur prior to and drive Aβ accumulation. The mechanisms underlying the genesis of hyperactivity remain to be determined but likely involve alterations in excitatory/inhibitory balance, which may be driven by either expression of the ApoE4 allele or direct or indirect loss of inhibitory control. This hyperactivity appears to play a central role underlying further cognitive decline in prodromal LOAD. Interactions between hyperactivity and amyloid and tau pathophysiological pathways will also require further study to conclusively determine mechanistic, causal relationships. The data described here support a role for all 3 in the disruption of network function and cognitive decline. Elucidation of those mechanisms will provide further insight into how age-dependent elevation of neural activity contributes to cognitive decline and progression of disease in LOAD. For example, resolution of the mechanistic, spatial, and temporal relationships between cortical Aβ and hippocampal dysfunction will be critical in guiding the timing and selection of appropriate therapeutic strategies.

Key evidence in support of this proposal is the efficacy of LEV, a therapy that effectively targets abnormal neural activity, in ameliorating cognitive decline in both preclinical animal models and human aMCI subjects. Based on findings from transgenic AD models regarding a role for neural activity in driving amyloid and tau pathology, we expect that targeting hyperactivity in aMCI will not only improve cognition, but may also slow or prevent the accumulation of pathology and clinical decline. While LEV is currently being pursued as a potential therapeutic for AD prevention in a human clinical trial, other therapeutic approaches that restore balanced neural activity may also prove effective in preventing disease progression and accumulating AD pathology. Successful development of such therapies could have vast implications for achieving therapeutic treatment in LOAD to reduce the prevalence of patients with AD dementia.

## Electronic supplementary material

Below is the link to the electronic supplementary material.

Required Author Forms Disclosure forms provided by the authors are available with the online version of this article. (PDF 1225 kb)
